# Impact of residual microcalcifcations on prognosis after neoadjuvant chemotherapy in breast cancer patients

**DOI:** 10.1186/s12905-024-02973-9

**Published:** 2024-03-20

**Authors:** Eun Young Kim, Kwan Ho Lee, Ji-Sup Yun, Yong Lai Park, Chan Heun Park, Sung Yoon Jang, Jai Min Ryu, Se Kyung Lee, Byung-Joo Chae, Jeong Eon Lee, Seok Won Kim, Seok Jin Nam, Jong Han Yu

**Affiliations:** 1 Department of Surgery, Kangbuk Samsung Hospital, Sungkyunkwan University School of Medicine, Seoul, Republic of Korea; 2 Division of Breast Surgery, Department of Surgery, Samsung Medical Center, Sungkyunkwan University School of Medicine, 81 Irwon-Ro, Gangnam-Gu, Seoul, 06351 Republic of Korea

**Keywords:** Breast neoplasms, Mammography, Microcalcification, Neoadjuvant, Recurrence

## Abstract

**Background:**

Residual microcalcifications after neoadjuvant chemotherapy (NAC) are challenging for deciding extent of surgery and questionable for impact on prognosis. We investigated changes in the extent and patterns of microcalcifications before and after NAC and correlated them with pathologic response. We also compared prognosis of patients depending on presence of residual microcalcifications after NAC.

**Methods:**

A total of 323 patients with invasive breast carcinoma treated with neoadjuvant chemotherapy at Kangbuk Samsung Hospital and Samsung Medical center from March 2015 to September 2018 were included. Patients were divided into four groups according to pathologic response and residual microcalcifications. Non-pCR_w/mic_ group was defined as breast non-pCR with residual microcalcifications. Non-pCR_w/o mic_ group was breast non-pCR without residual microcalcifications. pCR_w/mic_ group was breast pCR with residual microcalcifications. pCR_w/o mic_ group was breast pCR without residual microcalcifications. The first aim of this study is to investigate changes in the extent and patterns of microcalcifications before and after NAC and to correlate them with pathologic response. The second aim is to evaluate oncologic outcomes of residual microcalcifications according to pathologic response after NAC.

**Results:**

There were no statistical differences in the extent, morphology, and distribution of microcalcifications according to pathologic response and subtype after NAC (all *p* > 0.05). With a median follow-up time of 71 months, compared to pCR_w/o mic_ group, the hazard ratios (95% confidence intervals) for regional recurrence were 5.190 (1.160–23.190) in non-pCR_w/mic_ group and 5.970 (1.840–19.380) in non-pCR_w/o mic_ group. Compared to pCR_w/o mic_ group, the hazard ratios (95% CI) for distant metastasis were 8.520 (2.130–34.090) in non-pCR_w/mic_ group, 9.120 (2.850–29.200) in non-pCR_w/o mic_ group. Compared to pCR_w/o mic_, the hazard ratio (95% CI) for distant metastasis in pCR_w/mic_ group was 2.240 (0.230–21.500) without statistical significance (*p* = 0.486).

**Conclusions:**

Regardless of residual microcalcifications, patients who achieved pCR showed favorable long term outcome compared to non-pCR group.

**Supplementary Information:**

The online version contains supplementary material available at 10.1186/s12905-024-02973-9.

## Introduction

Neoadjuvant chemotherapy (NAC) is the standard treatment of locally advanced breast cancer. The indication for NAC has been extended to human epidermal growth factor receptor 2 (HER2)-positive and triple-negative breast cancer (TNBC) with clinical T stage ≥ 2 or N stage ≥ 1 due to developments in molecular pathology and targeted therapy. Although similar long-term outcomes were reported compared to adjuvant chemotherapy, NAC has many advantages including increased likelihood of breast conservation and prediction of sensitivity to chemotherapeutic agents. Pathologic complete response (pCR) is associated with better disease-free and overall survival [1, 2]. Although imaging modalities such as mammography, breast sonography, and magnetic resonance imaging (MRI) can predict the extent of a tumor, it is difficult to predict response when tumors are accompanied by microcalcifications.

Breast microcalcifications are calcium deposits within the breast tissue and can be classified according to the Breast Imaging Reporting and Data system (BI-RADS) developed by the American College of Radiology [3]. Most microcalcifications are manifestations of pure ductal carcinoma in situ (DCIS) or intraductal portions of invasive carcinomas [4]. Since DCIS rarely responds to systemic therapy and most microcalcifications do not decrease after NAC, partial mastectomy is often contraindicated even with radiological complete response (CR) on breast MRI [5, 6]. Decisions about surgical extent in patients with remaining extensive microcalcifications after NAC are challenging since residual microcalcifications show low correlation with pathologic response [7, 8]. The definition of pCR varies across studies [9]. Whether pCR is defined as ypTis or ypT0, eradication of residual invasive cancer of the breast is associated with excellent survival outcomes. Since most DCIS manifests as microcalcifications, eradication of all residual microcalcifications is questionable, and the impact of residual microcalcifications on long-term outcome remains unknown.

Therefore, the first aim of this study is to investigate changes in the extent and patterns of microcalcifications before and after NAC and to correlate them with pathologic response. The second aim is to evaluate oncologic outcomes of residual microcalcifications according to pathologic response after NAC.

## Methods

### Patients

A total of 323 patients with invasive breast carcinoma treated with neoadjuvant chemotherapy at Kangbuk Samsung Hospital and Samsung Medical center from March 2015 to September 2018 were included. Detailed information including patient age, tumor size, and clinical stage as well as estrogen receptor (ER), progesterone receptor, and HER2 positivity were extracted from the database. We excluded patients with stage IV breast cancer, without suspicious microcalcifications on mammography before and after NAC, or without available imaging results (mammography and/or MRI). This retrospective study was approved by the Institutional Review Board of Kangbuk Samsung Hospital (KBSMC 2018–09-033), and the requirement for individual informed consent was waived owing to the retrospective nature of the study. All methods were performed in accordance with the relevant guidelines.

### Assessment of mammography and MRI

All microcalcifications on mammography acquired pre- and post-NAC were reassessed by dedicated radiologists with 6–24 years of experience in breast imaging according to the BI-RADS lexicon [10]. Extent, morphology (amorphous, coarse heterogeneous, fine pleomorphic, fine linear or fine-linear branching), distribution (regional, grouped, linear, segmental, diffuse), and location of microcalcifications were evaluated. The categories of breast density were as follows: a. the breasts are almost entirely fatty. b. there are scattered areas of fibroglandular density. c. the breasts are heterogeneously dense, which may obscure small masses. d. the breasts are extremely dense, which lowers the sensitivity of mammography [10].

The extent of microcalcifications was measured in millimeters of the greatest dimensions in the craniocaudal (CC) and mediolateral oblique views. Multifocality was defined as multiple microcalcifications located in the same quadrant, whereas multicentricity was defined as microcalcifications located in more than one quadrant [11]. In cases of multifocal or multicentric microcalcifications, all lesions were individually measured, and the overall extent of disease, including all foci, was measured and used in analysis. “Decreased microcalcifications” was defined as a greater than 25% decrease in extent (not amount nor density) after NAC. “Increased microcalcifications” was defined as a greater than 25% increase in extent (not amount nor density) after NAC. "No change" referred to microcalcifications that neither increased nor decreased. “New” refers to suspicious microcalcifications newly developed after NAC [7]. Tumor response was assessed on MRI after completion of NAC, and the size of residual disease was categorized into four categories according to the response evaluation criteria in solid tumors (RECIST) and the Leeds modified response evaluation criteria in solid tumors, considering tumor size and enhancement characteristics [12, 13]. Radiological CR was defined as no residual enhancement within the original tumor bed or any residual mass. Partial response (PR) was defined as decrease in tumor diameter greater than 30% or improvement in the enhancement curve. Progressive disease (PD) was defined as an increase in the diameter of the tumor greater than 20%. Stable disease was defined as neither PR nor PD.

### Histopathologic analysis

Surgery was performed after completion of NAC. Specimens were sent to the mammography unit. Standard compression radiographs were obtained. Radiologists immediately confirmed the presence of microcalcifications inside the specimen. It was surgeon’s discretion to perform further resection of residual microcalcifications, in case specimen did not contain all microcalcifications. Board-certified pathologists with 10–24 years of experience reviewed breast specimens to estimate histopathologic tumor response to NAC. Breast pCR was defined as the absence of residual invasive carcinoma in resected breast specimens. Pathologic tumor response was categorized according to RECIST criteria [12, 14]. In cases of non-pCR, the largest histopathologic diameters of residual tumors were measured. Immunohistochemical staining was performed, and subtypes were determined according to ER, progesterone receptor, and HER2 status. Hormone receptor (HR) positivity was defined as ER and/or progesterone receptor positive.

### Survival outcome analysis

**Fig. 1:**
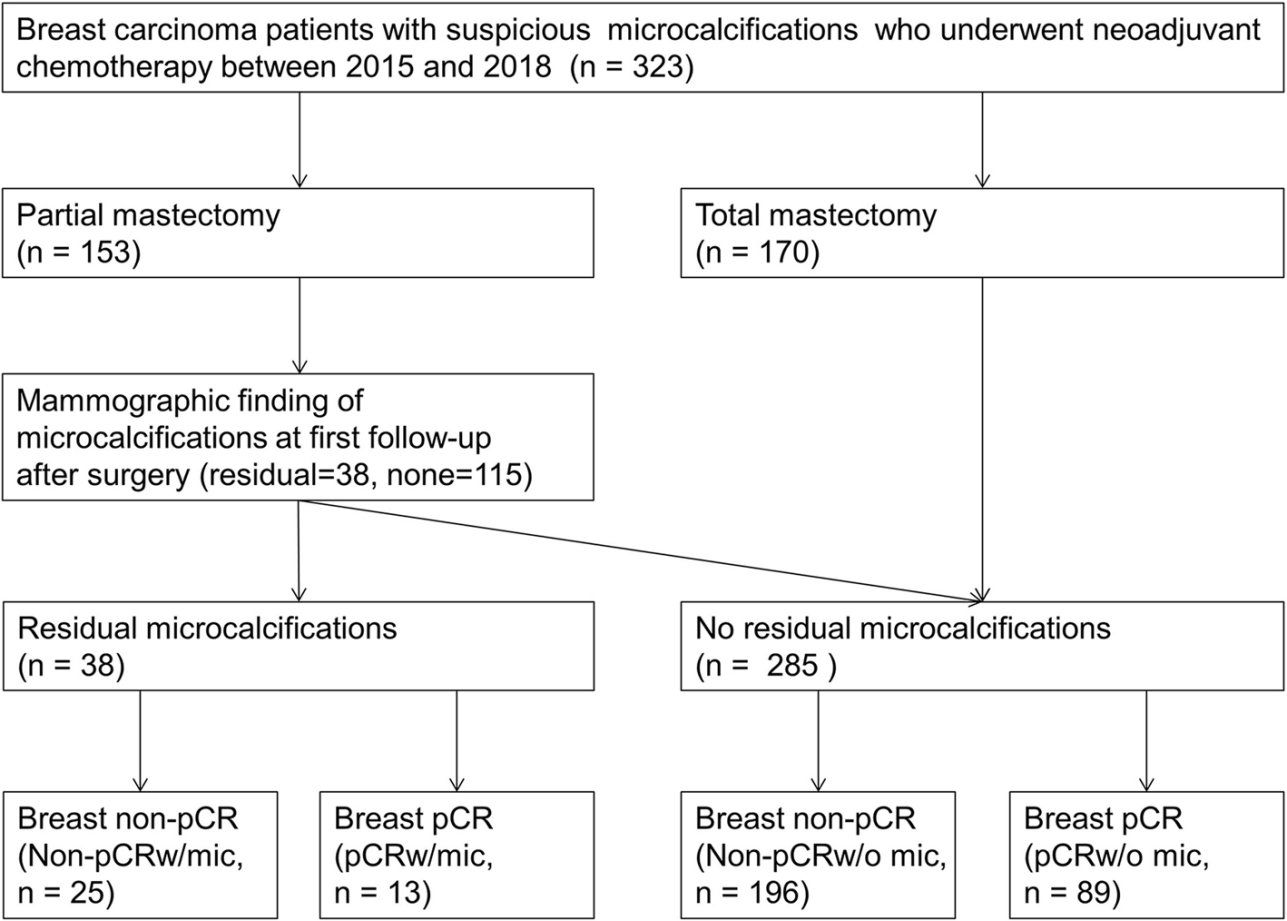
Flow chart of the selection process for study participants

Mammographic findings of microcalcifications were reassessed on first follow up after surgery (Fig. 1). Patients were classified into four groups according to pathologic response and residual microcalcifications. Non-pCR_w/mic_ (*n* = 25) was classified as breast non-pCR with residual microcalcifications. Non-pCR_w/o mic_ (*n* = 196) was classified as breast non-pCR without residual microcalcifications. pCR_w/mic_ (*n* = 13) was classified as breast pCR with residual microcalcifications. pCR_w/o mic_ (*n* = 89) was classified as breast pCR without residual microcalcifications.

Recurrence in any quadrant of the ipsilateral breast was defined as local recurrence. Recurrence in the ipsilateral breast, skin, chest wall, axillary, supraclavicular, internal mammary lymph nodes was defined as regional recurrence. Metastases in distant organs such as bone, lung, liver and brain were defined as distant metastases. Local relapse-free survival (LFS), regional relapse-free survival (RFS), and distant metastasis-free survival (DMFS) were calculated from date of breast cancer diagnosis to the respective event. Overall survival (OS) was defined from the date of breast cancer diagnosis to death from any cause. Patients who did not experience recurrence, distant metastasis, or death were censored at the last follow up.

### Statistical analysis

Data were summarized as frequencies and percentages. Descriptive data were tabulated. Changes in the extent, morphology, or distribution of microcalcifications after NAC were correlated using McNemar’s test. Changes in mammographic characteristics were correlated with pathologic response using Pearson's Chi-square test. Differences in tumor characteristics according to subtype were compared using Pearson's Chi-square test.

Univariate and multivariate logistic regression analyses were performed to identify variables predictive of breast pCR. The actuarial rates of recurrence and survival were calculated according to the Kaplan–Meier method, and comparisons were performed using the log-rank test. Univariate and multivariate analyses using the Cox proportional hazards regression model were used to determine predictive factors of recurrence and survival. Any missing data on mammographic characteristics were excluded from statistical analysis. Statistical analyses were performed using PASW software version 18.0 (IBM, Armonk, NY, USA). *P*-values < 0.05 were considered statistically significant.

## Results

A total of 323 patients was diagnosed with invasive breast carcinoma and underwent NAC between March 2015 and September 2018. The clinicopathologic characteristics of the study population before and after NAC are listed in Table 1. The median age of the study population was 47 years (range: 40–54 years). The median tumor size on MRI before NAC was 47.0 mm (interquartile range: 30.0–67.0 mm). Invasive ductal carcinoma, not otherwise specified, was the most common histologic type before NAC (*n* = 301, 93.2%). Other types included invasive lobular carcinoma (*n* = 10, 3.1%), mucinous (*n* = 2, 0.6%), and micropapillary (n = 1, 0.3%). The HR^−^/HER2^+^ subtype was noted in 31.3% (*n* = 101) of patients, and HR^−^/HER2^−^ was observed in 15.8% of patients (*n* = 51). Breast pCR (ypT0 and/or ypTis) was observed in 31.6% (*n* = 102) of patients. In 3.7% (*n* = 12) of patients, no residual in situ or invasive lesions (only ypT0) were seen.

**Table 1:** Clinicopathologic characteristics of the study population before and after neoadjuvant chemotherapy

Characteristics	Total (*n* = 323)	Non-pCR_w/ mic_ (*n* = 25)	Non-pCR_w/o mic_ (*n* = 196)	pCR_w/ mic_ (*n* = 13)	pCR_w/o mic_ (*n* = 89)	*P* value
Age, years	47 (40–54)	45 (37–55)	47 (39–54)	48 (39–56)	48 (41–52)	0.907
Pre-NAC histologic type						0.686
IDC	301 (93.2)	24 (96.0)	181 (92.3)	12 (92.3)	84 (94.4)	
ILC	10 (3.1)	1 (4.0)	7 (3.6)	0 (0)	2 (2.2)	
Mucinous	2 (0.6)	0 (0)	1 (0.5)	1 (7.7)	0 (0)	
Micropapillary	1 (0.3)	0 (0)	1 (0.5)	0 (0)	0 (0)	
Other	9 (2.8)	0 (0)	6 (3.1)	0 (0)	3 (3.4)	
Pre-NAC IHC subtype						< 0.001
HR + /HER2-	90 (27.9)	6 (24.0)	74 (37.8)	1 (7.7)	9 (10.1)	
HR + /HER2 +	81 (25.1)	5 (20.0)	43 (21.9)	3 (23.1)	30 (33.7)	
HR-/HER2 +	101 (31.3)	10 (40.0)	48 (24.5)	8 (61.5)	35 (39.3)	
HR-/HER2-	51 (15.8)	4 (16.0)	31 (15.8)	1 (7.7)	15 (16.9)	
Pre-NAC tumor size at MRI, mm	47 (30–67)	60 (33–72)	48 (32–67)	50 (27–55)	40 (30–60)	0.1
Pre-NAC Ki-67 ≥ 14%	278 (87.7)	22 (88.0)	161 (84.3)	11 (84.6)	84 (95.5)	0.069
NAC regimen						0.001
AC followed by T	118 (36.6)	6 (24.0)	88 (45.1)	0 (0)	24 (27)	
AC followed by TH	133 (41.3)	14 (56.0)	65 (33.3)	9 (69.2)	45 (50.6)	
TCHP	27 (8.4)	1 (4.0)	11 (5.6)	1 (7.7)	14 (15.7)	
AT	33 (10.2)	3 (12.0)	23 (11.8)	2 (15.4)	5 (5.6)	
Other	11 (3.4)	1 (4.0)	8 (4.1)	1 (7.7)	1 (1.1)	
Radiologic response on MRI						< 0.001
CR	74 (22.9)	2 (8.0)	22 (11.2)	9 (69.2)	41 (46.1)	
Non-CR	249 (77.1)	23 (92.0)	174 (88.8)	4 (30.8)	48 (53.9)	
Type of surgery						< 0.001
Total mastectomy	170 (52.6)	0 (0)	134 (68.4)	0 (0)	36 (40.4)	
Partial mastectomy	153 (47.4)	25 (100.0)	62 (31.6)	13 (100.0)	53 (59.6)	
Post-NAC tumor size at MRI, mm	18 (6–36)	16.50 (9.75–31.75)	22 (12.25–42.5)	0 (0–13)	7 (0–25)	< 0.001
Post-NAC histologic type						< 0.001
IDC	145 (45.2)	13 (52.0)	106 (54.1)	4 (30.8)	22 (25.8)	
DCIS	65 (20.1)	0 (0)	2 (1.0)	7 (53.8)	56 (62.9)	
ILC	99 (30.7)	12 (48.0)	87 (44.4)	0 (0)	0 (0)	
LCIS	1 (0.3)	0	0	0	1 (1.1)	
Other	1 (0.3)	0 (0)	1 (0.5)	0 (0)	0 (0)	
none (only ypT0)	12 (3.7)	0 (0)	0 (0)	2 (15.4)	10 (11.2)	
Breast pCR (ypT0 and/or ypTis)	102 (31.6)	0 (0)	0 (0)	13 (100)	89 (100)	< 0.001
Post-NAC tumor size (invasive), mm	16 (4.50–37)	12 (3–18)	21 (9–45)	0 (0–0)	0 (0–0)	< 0.001
Post-NAC tumor size (in situ), mm	24.50 (7–50)	35 (9.5–50)	30 (15–54.25)	20 (7.75–36)	10 (2–30)	0.002
Post-NAC extensive intraductal component	69 (28.2)	6 (26.1)	63 (32.3)	0 (0)	0 (0)	0.001
Post-NAC lymphovascular invasion	101 (34.1)	7 (28.0)	89 (45.4)	0 (0)	5 (7.5)	< 0.001
Post-NAC Ki-67 ≥ 14%	108 (50)	12 (63.2)	74 (48.7)	4 (80)	18 (45)	0.305
Follow-up (months)	74.6 (68.85–86.44)	72.7 (69.39–78.26)	75.2 (68.29–84.73)	71.8 (68.99–86.37)	74.7 (68.93–87.19)	0.816
Local recurrence	39 (12.1)	5 (20.0)	31 (15.8)	0 (0)	3 (3.4)	0.007
Regional recurrence	43 (13.3)	4 (16.0)	36 (18.4)	0 (0)	3 (3.4)	0.003
Distant metastasis	62 (19.2)	6 (24.0)	52 (26.5)	1 (7.7)	3 (3.4)	< 0.001
Death	38 (11.8)	4 (16.0)	31 (15.8)	0 (0)	3 (3.4)	0.010

Data are presented as median (interquartile range) or number (%)

*Abbreviations*: *NAC* Neoadjuvant chemotherapy, *IDC* Invasive ductal carcinoma, *ILC* Invasive lobular carcinoma, *DCIS* Ductal carcinoma in situ, *HR* Hormone receptor, *HER2* Human epidermal growth factor receptor 2, *AC* Adriamycin/cyclophosphamide, *CR* Complete response, *T* Docetaxel, *TH* Docetaxel/trastuzumab, *TCHP* Docetaxel/carboplatin/trastuzumab/pertuzumab, *AT* Adriamycin/docetaxel, *LCIS* Lobular carcinoma in situ, *pCR* Pathologic complete response

Mammographic findings before and after NAC are described in Supplementary Table 1. Multifocal/multicentric microcalcifications were noted in 110 patients (34.1%) before NAC. Our results showed that 126 (39.3%) and 148 (45.7%) of patients had pleomorphic microcalcifications before and after NAC, respectively. In addition, 69 (21.3%) and 74 (22.9%) patients showed fine linear/linear branching microcalcifications before and after NAC, respectively. After NAC, there was no change in extent of microcalcifications in 221 (68.4%) patients. Decrease of extent was noted in 74 (23.0%) patients, while increase was evident in 18 (5.6%) patients. The median extent of microcalcifications was 41.0 [interquartile range (IQR) 25.0–66.5] mm before NAC and 43.0 (IQR 22.0–70.0) mm after NAC. The majority of microcalcifications did not present changes in morphology (72.5%) after NAC.

Changes in mammographic and MRI characteristics after NAC according to pathologic response are described in Supplementary Table 2. Changes in extent, morphology of microcalcifications, and mammographic density after NAC did not differ significantly according to pathologic response (all *p* > 0.05). Changes in sizes of tumors measured on MRI after NAC were significantly different between the pCR and non-pCR groups (*p* < 0.001). Fifty patients (49.0%) in the pCR group showed radiological CR on MRI after NAC. In contrast, 153 patients (69.2%) in the non-pCR group showed PR and 21 patients (9.5%) in the non-pCR group exhibited SD on MRI. This implies that changes in size of tumor on MRI was correlated with pathologic response, however changes in the extent and morphology of microcalcifications after NAC were not correlated with pathologic response.

**Fig. 2:**
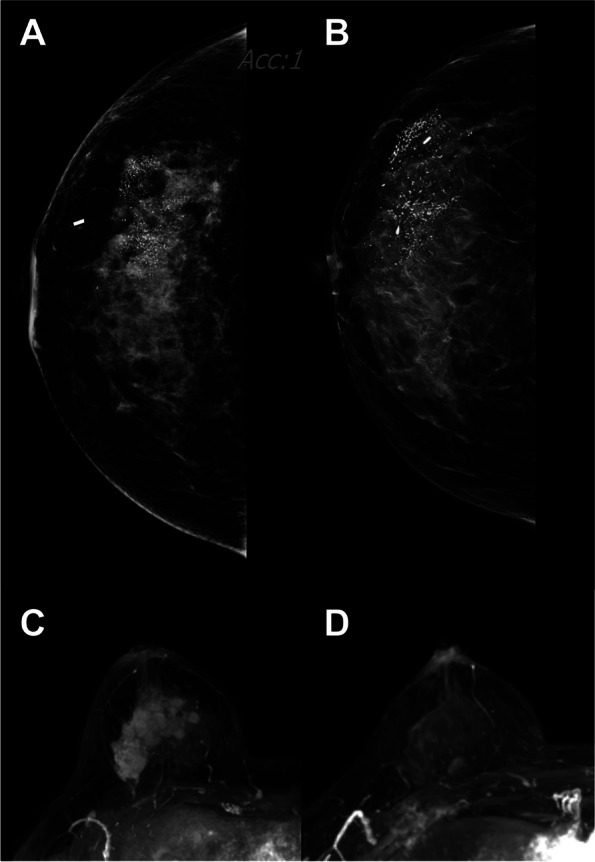
A 44-year-old woman with 53-mm HR^−^/HER2.^+^ subtype in the upper outer quadrant of the right breast. Before NAC, **A** CC image of mammography showed a 50-mm extent of linear branching microcalcifications. After completion of NAC, **B** the size and density of the mass were decreased, but the extent of microcalcifications did not decrease on mammography. **C** A maximum intensity projection (MIP) image from MRI revealed a 55-mm heterogeneous mass with segmental enhancement to the nipple. After NAC, **D** the MIP image from MRI revealed only small enhancing foci in the upper breast. The patient underwent total mastectomy, and no residual invasive or in situ component was noted. This patient achieved pCR (ypT0)

**Fig. 3:**
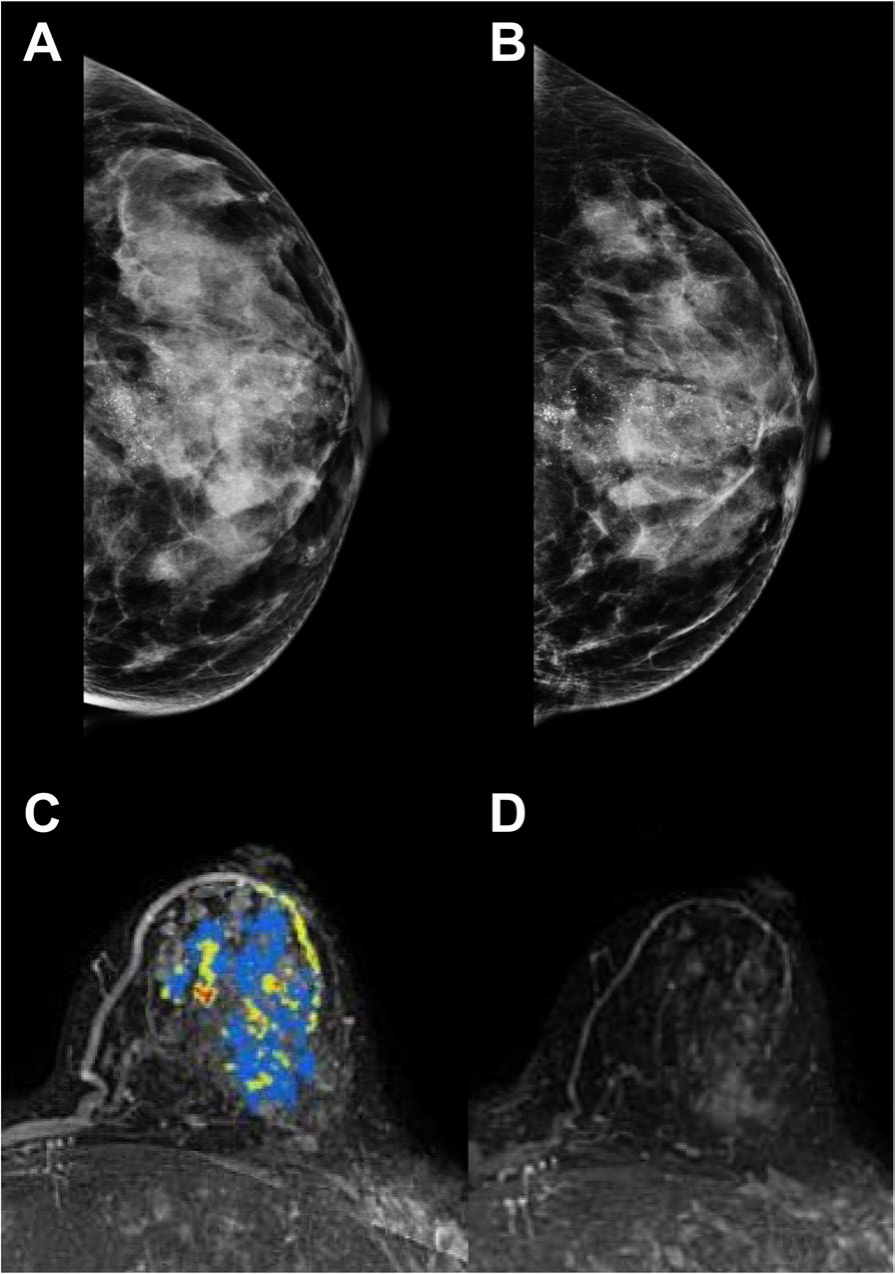
A 52-year-old woman with 44-mm HR^+^/HER2^−^ subtype in the lower central quadrant of the left breast. Before NAC, **A** a CC image of mammography revealed a 62-mm extent of segmental distribution of microcalcifications. After NAC, **B** the size, mass, and extent of microcalcifications did not decrease on mammography. On **C** a computer-aided detection (CAD) image from MRI, a 44-mm, irregularly shaped, non-mass-like enhancement was noted. After NAC, **D** the CAD image from MRI revealed that the enhancement had decreased to a 35-mm extent. She underwent total mastectomy, and the pathology showed a residual DCIS of 42 mm

Changes in mammographic and MRI characteristics after NAC differed according to subtype (Table 2; Figs. 2 and 3). The extent of microcalcifications after NAC was largest in HR^−^ HER2^+^ subtype and smallest in HR^−^ HER2^−^ subtype (48.0 mm vs. 23.5 mm, *p* = 003). There were no differences in changes in extent of microcalcifications according to subtype (*p* = 0.147), though there was a statistically significant difference in the change in morphology of microcalcifications after NAC according to subtype (*p* < 0.001). About 22–24% of the HR^+^ HER2^+^ and HR^−^ HER2^+^ subtypes showed changes in morphology of microcalcifications. In addition, the pathologic response differed according to subtype (*p* < 0.001). HR^−^ HER2^+^ subtype showed the highest pCR rate (42.6%), and the HR^+^ HER2^−^ subtype showed the lowest pCR rate (11.1%).

**Table 2:** Changes in mammographic and MRI characteristics after NAC according to subtype

	**HR**^**+**^** HER2**^**−**^** (*n* = 90)**	**HR**^**−**^** HER2**^**−**^** (*n* = 51)**	**HR**^**+**^** HER2**^**+**^** (*n* = 81)**	**HR**^**−**^** HER2**^**+**^** (*n* = 101)**	***P* value**
Post-NAC tumor size (invasive), mm	25.0 (12.0, 50.0)	19.5 (6.3, 33.5)	13.0 (3.3, 25.0)	14.0 (3.3, 25.0)	0.004
Post-NAC tumor size (in situ), mm	30.0 (11.5, 60.0)	25.0 (7.0, 40.0)	20.0 (5.3, 46.5)	25.0 (6.0, 43.5)	0.346
Post-NAC extent of microcalcifications, mm	44.0 (27.0, 71.0)	23.5 (13.3, 35.8)	41.0 (20.5, 70.0)	48.0 (28.8, 70.3)	0.003
Changes in extent of microcalcifications					0.147
No change	60 (66.7)	31 (60.8)	50 (61.7)	67 (66.3)	
Decreased	18 (20.0)	8 (15.7)	19 (23.5)	25 (24.8)	
Increased	5 (5.6)	2 (3.9)	6 (7.4)	4 (4.0)	
New	2 (2.2)	2 (3.9)	1 (1.2)	4 (4.0)	
Not specified	5 (5.6)	8 (15.7)	5 (6.2)	1 (1.0)	
Change in morphology of microcalcifications					< 0.001
No change	46 (51.1)	18 (35.3)	35 (43.2)	51 (50.5)	
Change	14 (15.6)	1 (2.0)	20 (24.7)	22 (21.8)	
Not specified	30 (33.3)	32 (62.7)	26 (32.1)	28 (27.7)	
Radiologic response of breast cancer on MRI					0.073
CR	11 (12.2)	10 (19.6)	26 (32.1)	27 (26.7)	
Non-CR	78 (86.7)	41 (80.4)	54 (66.7)	73 (72.3)	
Not specified	1 (1.1)	0 (0.0)	1 (1.2)	1 (1.0)	
Pathologic response of breast cancer					< 0.001
pCR	10 (11.1)	16 (31.4)	33 (40.7)	43 (42.6)	
Non-pCR	80 (88.9)	35 (68.6)	48 (59.3)	58 (57.4)	

Data are mean (standard deviation) or number (percentage)

*Abbreviations*: *NAC* Neoadjuvant chemotherapy, *HR* Hormone receptor, *HER2* Human epidermal growth factor receptor 2, *CR* Complete response, *pCR* Pathologic complete response

Predictive factors of pCR (ypT0 or ypTis) are listed in Supplementary Table 3. The univariate-adjusted odds ratio for pCR was 3.046 (95% confidence interval [CI]: 1.475–6.290) for HER2^+^ subtype, 5.091 (95% CI: 2.900–8.939) for tumor size < 10 mm on MRI after NAC, 41.667 (95% CI: 5.276–329.047) for radiologic CR on MRI, and 2.824 (95% CI: 1.734–4.598) for partial mastectomy. Only HER2^+^ subtype remained significant after multivariate analysis. Changes in extent and morphology of microcalcifications were not predictive factors in either univariate or multivariate regression.

Univariate and multivariate analyses were performed to identify variables for LFS, RFS, DMFS, and OS (Tables 3, 4, 5 and 6). HER2^+^ positivity showed negative correlations with local and regional recurrence, and lymphovascular invasion and Ki-67 ≥ 14% after NAC were positively correlated with local and regional recurrence, as well as DMFS and OS. Other factors including extent of microcalcifications, presence of residual microcalcifciaiton (regardless of pCR) after NAC did not predict recurrence or survival. Since there were only few cases of local, regional recurrences, distant metastasis and death in patients who achieved radiologic CR on MRI, it was difficult to calculate odds ratio for the variable “radiologic response of MRI” in multivariate analysis. We could not provide odds ratio for that variable in Tables 3, 4, 5 and 6.

**Table 3:** Univariate and multivariate analyses of local recurrence

Characteristics	Univariate		Multivariate	
	**Odds ratio (95% CI)**	***P* value**	**Odds ratio (95% CI)**	***P* value**
Age at diagnosis, years (ref = < 40)				
≥ 40	0.712 (0.350–1.530)	0.363		
Clinical T stage before NAC (ref = cT1)				
cT2-4	1.387 (0.250- 25.810)	0.758		
Clinical N stage before NAC (ref = cT0)				
cN1-3	1.232 (0.340–7.950)	0.785		
Lymphovascular invasion (ref = absent)				
Present	6.416 (3.110- 14.090)	< 0.001	5.950 (2.430–16.130)	< 0.001
HR + (ref = negative)				
Positive	0.459 (0.220–0.910)	0.028	0.490 (0.170–1.300)	0.159
HER2^+^(ref = negative)				
Positive	0.462 (0.210- 0.970)	0.046	0.340 (0.130- 0.860)	0.026
Ki-67 before NAC (ref = < 14%)				
≥ 14%	1.728 (0.580- 7.430)	0.383		
Ki-67 after NAC (ref = < 14%)				
≥ 14%	4.810 (2.090- 12.500)	< 0.001	5.010 (1.760- 16.120)	0.004
Radiologic response of MRI (ref = CR)				
Non-CR	6.283 (1.860–39.240)	0.013	N/A	0.988
Extent of surgery (ref = total mastectomy)				
Partial mastectomy	0.661 (0.330–1.300)	0.237		
Pre-NAC extent of microcalcifications, mm (ref = < 10)				
≥ 10	0.500 (0.120–3.440)	0.397		
Post-NAC extent of microcalcifications, mm (ref = < 10)				
≥ 10	0.590 (0.140- 3.990)	0.512		
Residual microcalcification (ref = absent)				
Present	1.119 (0.364–2.839)	0.827	1.740 (0.410–6.410)	0.423

*Abbreviations*: *CI* Confidence interval, *HER2* Human epidermal growth factor receptor 2, *NAC* Neoadjuvant chemotherapy, *N/A* Not applicable, *CR* Complete response, *PR* Partial response, *ref* Reference, *SD* Stable disease, *PD* Progressive disease

**Table 4:** Univariate and multivariate analyses of regional recurrence

Characteristics	Univariate		Multivariate	
	**Odds ratio (95% CI)**	***P*** value	**Odds ratio (95% CI)**	***P*** value
Age at diagnosis, years (ref = < 40)				
≥ 40	0.642 (0.320 -1.320)	0.211		
Clinical T stage before NAC (ref = cT1)				
cT2-4	1.556 (0.290- 28.910)	0.677		
Clinical N stage before NAC (ref = cT0)				
cN1-3	1.390 (0.380 -8.950)	0.667		
Lymphovascular invasion (ref = absent)				
Present	7.758 (3.810- 16.910)	< 0.001	6.000 (2.510- 15.650)	< 0.001
HR + (ref = negative)				
Positive	0.606 (0.310- 1.160)	0.131		
HER2^+^(ref = negative)				
Positive	0.373 (0.170- 0.770)	0.009	0.280 (0.110- 0.690)	0.007
Ki-67 before NAC (ref = < 14%)				
≥ 14%	1.958 (0.660- 8.390)	0.282		
Ki-67 after NAC (ref = < 14%)				
≥ 14%	4.808 (2.180 -11.790)	< 0.001	7.940 (3.050- 23.400)	< 0.001
Radiologic response of MRI (ref = CR)				
Non-CR	14.812 (3.140- 264.940)	0.008	N/A	0.988
Extent of surgery (ref = total mastectomy)				
Partial mastectomy	0.490 (0.240- 0.950)	0.039	1.400 (0.510- 3.800)	0.504
Pre-NAC extent of microcalcifications, mm (ref = < 10)				
≥ 10	0.545 (0.130- 3.740)	0.458		
Post-NAC extent of microcalcifications, mm (ref = < 10)				
≥ 10	0.619 (0.150- 4.180)	0.550		
Residual microcalcification (ref = absent)				
Present	0.742 (0.213–1.992)	0.592	0.600 (0.100- 2.880)	0.543

*Abbreviations*: *CI* Confidence interval, *HER2* Human epidermal growth factor receptor 2, *NAC* Neoadjuvant chemotherapy, *N/A* Not applicable, *CR* Complete response, *PR* Partial response, *ref* Reference, *SD* Stable disease, *PD* Progressive disease

**Table 5:** Univariate and multivariate analyses of distant metastasis

Characteristics	Univariate		Multivariate	
	**Odds ratio (95% CI)**	***P*** value	**Odds ratio (95% CI)**	***P*** value
Age at diagnosis, years (ref = < 40)				
≥ 40	0.694 (0.380- 1.300)	0.241		
Clinical T stage before NAC (ref = cT1)				
cT2-4	1.071 (0.270- 7.150)	0.931		
Clinical N stage before NAC (ref = cT0)				
cN1-3	2.166 (0.600- 13.870)	0.309		
Lymphovascular invasion (ref = absent)				
Present	6.594 (3.610- 12.450)	< 0.001	4.590 (2.230- 9.830)	< 0.001
HR + (ref = negative)				
Positive	0.878 (0.500- 1.530)	0.644		
HER2^+^(ref = negative)				
Positive	0.516 (0.270- 0.960)	0.038	0.530 (0.250- 1.110)	0.099
Ki-67 before NAC (ref = < 14%)				
≥ 14%	1.357 (0.580- 3.740)	0.515		
Ki-67 after NAC (ref = < 14%)				
≥ 14%	2.606 (1.390- 5.050)	0.004	3.230 (1.520- 7.160)	0.003
Radiologic response of MRI (ref = CR)				
Non-CR	11.429 (3.440- 70.820)	0.001	N/A	0.988
Extent of surgery (ref = total mastectomy)				
Partial mastectomy	0.546 (0.300- 0.960)	0.039	1.400 (0.610- 3.210)	0.426
Pre-NAC extent of microcalcifications, mm (ref = < 10)				
≥ 10	0.891 (0.210- 6.050)	0.887		
Post-NAC extent of microcalcifications, mm (ref = < 10)				
≥ 10	1.056 (0.260- 7.050)	0.946		
Residual microcalcification (ref = absent)				
Present	0.944 (0.366–2.145)	0.897	0.810 (0.200–2.920)	0.755

*Abbreviations*: *CI* Confidence interval, *HER2* Human epidermal growth factor receptor 2, *NAC* Neoadjuvant chemotherapy, *N/A* Not applicable, *CR* Complete response, *PR* Partial response, *ref* Reference, *SD* Stable disease, *PD* Progressive disease

**Table 6:** Univariate and multivariate analyses of death

Characteristics	Univariate		Multivariate	
	**Odds ratio (95% CI)**	***P*** value	**Odds ratio (95% CI)**	***P*** value
Age at diagnosis, years (ref = < 40)				
≥ 40	0.788 (0.380- 1.730)	0.534		
Clinical T stage before NAC (ref = cT1)				
cT2-4	1.345 (0.250- 25.040)	0.780		
Clinical N stage before NAC (ref = cT0)				
cN1-3	0.727 (0.230- 3.230)	0.625		
Lymphovascular invasion (ref = absent)				
Present	7.096 (3.380- 16.050)	< 0.001	5.230 (2.190- 13.620)	< 0.001
HR + (ref = negative)				
Positive	0.482 (0.230 -0.960)	0.041	0.420 (0.150- 1.110)	0.082
HER2^+^(ref = negative)				
Positive	0.436 (0.200- 0.910)	0.031	0.320 (0.120- 0.780)	0.015
Ki-67 before NAC (ref = < 14%)				
≥ 14%	1.672 (0.560- 7.190)	0.413		
Ki-67 after NAC (ref = < 14%)				
≥ 14%	4.167 (1.870- 10.270)	0.001	4.030 (1.480- 12.090)	0.009
Radiologic response of MRI (ref = CR)				
Non-CR	12.741 (2.680- 228.220)	0.013	N/A	0.988
Extent of surgery (ref = total mastectomy)				
Partial mastectomy	0.613 (0.300- 1.220)	0.169		
Pre-NAC extent of microcalcifications, mm (ref = < 10)				
≥ 10	0.433 (0.100- 2.990)	0.309		
Post-NAC extent of microcalcifications, mm (ref = < 10)				
≥ 10	0.533 (0.130- 3.620)	0.436		
Residual microcalcification (ref = absent)				
Present	0.869 (0.248–2.351)	0.801	1.670 (0.400–6.010)	0.447

*Abbreviations*: *CI* Confidence interval, *HER2* Human epidermal growth factor receptor 2, *NAC* Neoadjuvant chemotherapy, *N/A* Not applicable, *CR* Complete response, *PR* Partial response, *ref* Reference, *SD* Stable disease, *PD* Progressive disease

**Fig. 4:**
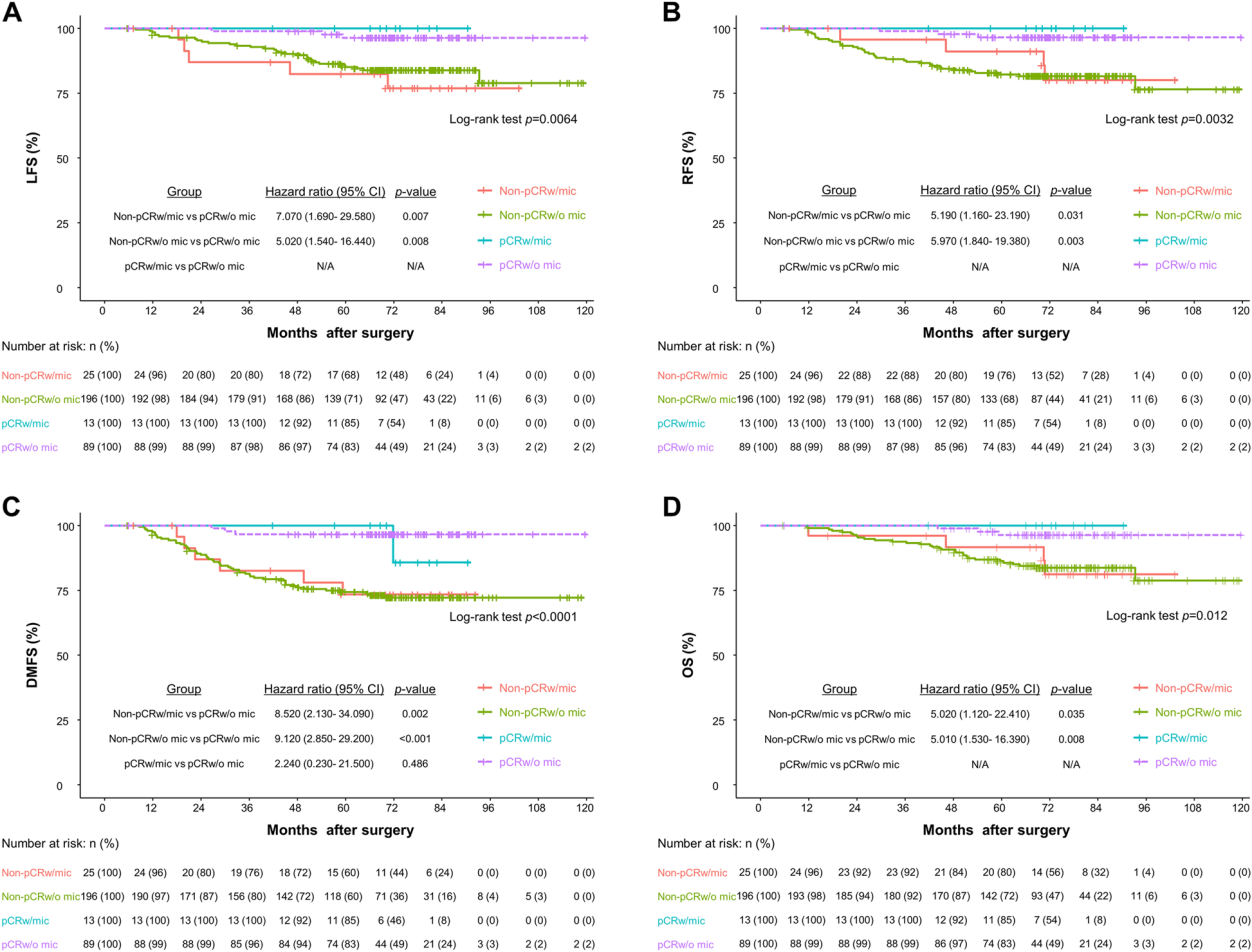
Kaplan–Meier curves of survival outcomes stratified according to pathologic response and residual microcalcifications status. The Kaplan–Meier curves of LFS, RFS, DMFS, and OS are shown (**a**–**d**). The hazard ratio was calculated by univariate Cox regression analysis

We evaluated long-term outcomes of residual microcalcifications after NAC according to pathologic response (Fig. 4a-d) (See Supplementary Table 4). The median follow-up period was 74.6 months. Compared to pCR_w/o mic_, the hazard ratios (95% CI) for regional recurrence in non-pCR_w/mic_ group, non-pCR_w/o mic_ group were 5.190 (1.160–23.190), 5.970 (1.840–19.380), respectively. Compared to pCR_w/o mic_, the hazard ratios (95% CI) for distant metastasis in non-pCR_w/mic_ group, non-pCR_w/o mic_ group were 8.520 (2.130–34.090), 9.120 (2.850–29.200), respectively. Compared to pCR_w/o mic_, the hazard ratio (95% CI) for distant metastasis in pCR_w/mic_ group was 2.240 (0.230–21.500) without statistical significance (*p* = 0.486). We could not perform statistical analysis for LFS, RFS, OS in pCR_w/ mic_ group owing to small number of events. This association was consistently observed in terms of LFS and OS, in which non-pCR were associated with an increased risk regardless of residual microcalcifications.

## Discussion

In the present study, we aimed to determine whether residual microcalcifications reflect the chemotherapeutic response of breast cancer and to clarify whether changes in the characteristics of microcalcifications after NAC predict pathologic response and affect long-term outcomes. We demonstrated that extent of residual microcalcifications after NAC were not correlated with extent of residual cancer. Only HER2^+^ subtype and radiologic CR on MRI were predictors of pCR.

Past studies demonstrated no correlation between changes in microcalcifications and pCR [15, 16] and recommended excision of indeterminate or suspicious-looking microcalcifications after NAC [17]. Yim et al. found that changes in microcalcifications after NAC were correlated with tumor response to NAC [18]. They reported more frequent decreases in microcalcifications in cancers showing CR on MRI or Miller-Payne grade 5 and more frequent increases of microcalcifications in cancers showing PD on MRI or Miller-Payne grade 1. They insisted upon complete removal of all microcalcifications since residual microcalcifications can be problematic as they increase in number on subsequent mammograms. In contrast, some authors have argued that residual microcalcifications are not always indications for total mastectomy. Some insisted that decisions about surgical methods should rely on immunohistochemical subtypes and evidence of radiologic CR on MRI. Mazari et al. reported that patients with HER2^+^ subtype can achieve pCR even with residual mammographic microcalcifications [19]. This result is not surprising since higher pCR rate in the HER2^+^ subtype was reported in past trials such as the NeoSphere and TRYPHAENA studies [20, 21]. In our study, we also found that radiologic response on MRI and immunohistochemical subtype were correlated with pCR. However, changes in the extent and distribution of microcalcifications were not correlated with pathologic response. Because of these conflicting results, there is no consensus regarding management of residual microcalcifications after NAC.

Currently, there is no standardized definition of pCR. Some studies have included noninvasive cancer residuals [14], whereas others have identified pCR as complete eradication of all invasive and noninvasive cancer [22]. It remains questionable whether patients with residual noninvasive cancer after NAC should be considered as having achieved pCR. Von Minckwitz et al. proposed that pCR should be defined only as no invasive and no in situ residuals in breasts and nodes since these discriminate favorable and unfavorable outcomes [2]. Others argued that a residual DCIS component is not a worse prognosis than complete eradication of DCIS [23–25]. Thus, it is unclear, in terms of prognosis such as recurrence or survival, whether all microcalcifications should be eradicated since they can be correlated with residual DCIS. Some researchers insist that malignant microcalcifications are more strongly associated with invasive breast carcinomas since they contain larger hydroxyapatite particles [26]. However, such reports solely reported cases of microcalcifications without NAC. Kim et al. reported that 37.5% of residual microcalcifications (36/96) were associated with only in situ component in final pathology after NAC [8]. They further stated that the extent of microcalcifications on mammography after NAC did not correlate with the extent of residual cancer in 38.5% of the patients. Further research is necessary to analyze the subgroup of patients who are likely to achieve pCR regardless of the extent of residual microcalcifications.

Residual microcalcifications with pCR in the breast do not affect long-term outcomes. In our study, 102 (31.6%) achieved breast pCR, 65 of whom had residual DCIS. Of the patients with breast pCR, there were four cases of distant metastases. Two patients from the pCR_w/o mic_ group developed simultaneous local recurrence and metastatic disease eventually leading to death. One patient in the pCR_w/o mic_ group developed distant recurrence as a first event, eventually leading to death. One patient from the pCR_w/mic_ group developed distant metastases. With small number of patients and few events in each group, this study is underpowered to detect anything but a difference in outcome between the non-pCR and pCR groups. Consequently, we reached no definitive conclusion regarding difference in long term outcome between pCR_w/o mic_ group and pCR_w/mic_ group.

In contrast to our results, JY Lee et al. found that extent of surgery in cases of tumor regression without change in extent of microcalcifications must be determined based on residual microcalcifications [27]. In their study, five (10.2%) patients in the RESMIN (reduced mass with no change in residual microcalcifications) group experienced locoregional recurrence, and 80% reported residual cancers. In line with our study, JH Cheun et al. reported that margin involvement did not affect the risk of local recurrence in patients showing pCR with residual DCIS in the breast [28]. They demonstrated that the prognosis of patients with residual DCIS was not significantly affected by resection margin status, concluding that residual DCIS after NAC is clinically and pathologically different from usual DCIS. Thus, there may be no need to worry about residual DCIS on resection margin.

There are several limitations of our study. First, it was a retrospectively designed multicenter study that could involve bias owing to the heterogeneity of data. For example, the decision to perform partial mastectomy with remaining microcalcifications depended on surgeon preference. Therefore, large prospective randomized trials are needed. Second, we did not assess other types of pathologic responses such as residual cancer burden [29], grading by Miller-Payne [30], or CPS + EG score [31]. However, Miller-Payne grade evaluates tumor response based on reduction of tumor cellularity not the size of invasive or in situ carcinoma [30], and CPS + EG score does not include information of the invasive or in situ component. Therefore, the commonly used yp TNM stage by AJCC used in this study is a reasonable criterion to assess pathologic response. Last, we could not correlate residual microcalcifications with histopathological findings of residual tumours whether these microcalcifications were correlated with benign, in situ or invasive component. Prospective study is needed to find out the correlation between microcalcifications and histopathology in the future.

In conclusion, changes in the extent of microcalcifications were not correlated with pathologic response. HER2^+^ subtype and post-NAC findings on MRI were significant predictive factors for pCR. In patients who achieved pCR, residual microcalcifications did not increase the risk of recurrence, distant metastases, or death. Until standard guidelines are established, the eradication of residual microcalcifications remains challenging.

### Supplementary Information

**Supplementary Material 1.**

**Supplementary Material 2.**

**Supplementary Material 3.**

**Supplementary Material 4.**

## Data Availability

All data used by or generated in this study is available from the corresponding author upon reasonable request.

## References

[1] Cortazar P, Zhang L, Untch M, Mehta K, Costantino JP, Wolmark N (2014). Pathological complete response and long-term clinical benefit in breast cancer: the CTNeoBC pooled analysis. Lancet.

[2] von Minckwitz G, Untch M, Blohmer JU, Costa SD, Eidtmann H, Fasching PA (2012). Definition and impact of pathologic complete response on prognosis after neoadjuvant chemotherapy in various intrinsic breast cancer subtypes. J Clin Oncol.

[3] Cox RF, Morgan MP (2013). Microcalcifications in breast cancer: lessons from physiological mineralization. Bone.

[4] Kopans DB (2007). Interpreting the mammogram. Breast imaging.

[5] Doebar SC, van den Broek EC, Koppert LB, Jager A, Baaijens MHA, Obdeijn IAM (2016). Extent of ductal carcinoma in situ according to breast cancer subtypes: a population-based cohort study. Breast Cancer Res Treat.

[6] Holmes D, Colfry A, Czerniecki B, Dickson-Witmer D, Francisco Espinel C, Feldman E (2015). Performance and practice Guideline for the Use of Neoadjuvant systemic therapy in the management of breast Cancer. Ann Surg Oncol.

[7] Adrada BE, Huo L, Lane DL, Arribas EM, Resetkova E, Yang W (2015). Histopathologic correlation of residual mammographic microcalcifications after neoadjuvant chemotherapy for locally advanced breast cancer. Ann Surg Oncol.

[8] Kim EY, Do SI, Yun JS, Park YL, Park CH, Moon JH (2020). Preoperative evaluation of mammographic microcalcifications after neoadjuvant chemotherapy for breast cancer. Clin Radiol.

[9] Pennisi A, Kieber-Emmons T, Makhoul I, Hutchins L (2016). Relevance of Pathological Complete Response after neoadjuvant therapy for breast Cancer. Breast Cancer (Auckl).

[10] Committee BI-RADS, American College of Radiology (2013). ACR BI-RADS® atlas: breast imaging reporting and data system.

[11] Zhou MR, Tang ZH, Li J, Fan JH, Pang Y, Yang HJ (2013). Clinical and pathologic features of multifocal and multicentric breast cancer in Chinese women: a retrospective cohort study. J Breast Cancer.

[12] Eisenhauer EA, Therasse P, Bogaerts J, Schwartz LH, Sargent D, Ford R (2009). New response evaluation criteria in solid tumours: revised RECIST guideline (version 1.1). Eur J Cancer.

[13] Fatayer H, Sharma N, Manuel D, Kim B, Keding A, Perren T (2016). Serial MRI scans help in assessing early response to neoadjuvant chemotherapy and tailoring breast cancer treatment. Eur J Surg Oncol.

[14] Green MC, Buzdar AU, Smith T, Ibrahim NK, Valero V, Rosales MF (2005). Weekly paclitaxel improves pathologic complete remission in operable breast cancer when compared with paclitaxel once every 3 weeks. J Clin Oncol.

[15] Feliciano Y, Mamtani A, Morrow M, Stempel MM, Patil S, Jochelson MS (2017). Do calcifications seen on Mammography after Neoadjuvant Chemotherapy for breast Cancer always need to be excised?. Ann Surg Oncol.

[16] Weiss A, Lee KC, Romero Y, Ward E, Kim Y, Ojeda-Fournier H (2014). Calcifications on mammogram do not correlate with tumor size after neoadjuvant chemotherapy. Ann Surg Oncol.

[17] Morrow M, Khan AJ (2020). Locoregional Management after Neoadjuvant Chemotherapy. J Clin Oncol.

[18] Yim H, Ha T, Kang DK, Park SY, Jung Y, Kim TH (2019). Change in microcalcifications on mammography after neoadjuvant chemotherapy in breast cancer patients: correlation with tumor response grade and comparison with lesion extent. Acta Radiol.

[19] Mazari FAK, Sharma N, Dodwell D, Horgan K (2018). Human epidermal growth factor 2-positive breast Cancer with Mammographic Microcalcification: relationship to Pathologic Complete Response after Neoadjuvant Chemotherapy. Radiology.

[20] Gianni L, Pienkowski T, Im YH, Tseng LM, Liu MC, Lluch A (2016). 5-year analysis of neoadjuvant pertuzumab and trastuzumab in patients with locally advanced, inflammatory, or early-stage HER2-positive breast cancer (NeoSphere): a multicentre, open-label, phase 2 randomised trial. Lancet Oncol.

[21] Schneeweiss A, Chia S, Hickish T, Harvey V, Eniu A, Hegg R (2013). Pertuzumab plus Trastuzumab in combination with standard neoadjuvant anthracycline-containing and anthracycline-free chemotherapy regimens in patients with HER2-positive early breast cancer: a randomized phase II cardiac safety study (TRYPHAENA). Ann Oncol.

[22] von Minckwitz G, Rezai M, Loibl S, Fasching PA, Huober J, Tesch H (2010). Capecitabine in addition to anthracycline- and taxane-based neoadjuvant treatment in patients with primary breast cancer: phase III GeparQuattro study. J Clin Oncol.

[23] Mazouni C, Peintinger F, Wan-Kau S, Andre F, Gonzalez-Angulo AM, Symmans WF (2007). Residual ductal carcinoma in situ in patients with complete eradication of invasive breast cancer after neoadjuvant chemotherapy does not adversely affect patient outcome. J Clin Oncol.

[24] Jones RL, Lakhani SR, Ring AE, Ashley S, Walsh G, Smith IE (2006). Pathological complete response and residual DCIS following neoadjuvant chemotherapy for breast carcinoma. Br J Cancer.

[25] Mazouni C, Peintinger F, Wan-Kau S, Andre F, Gonzalez-Angulo AM, Symmans F (2007). Effect on patient outcome of residual DCIS in patients with complete eradication of invasive breast cancer after neoadjuvant chemotherapy. J Clin Oncol.

[26] Karamouzis MV, Likaki-Karatza E, Ravazoula P, Badra FA, Koukouras D, Tzorakoleftherakis E (2002). Non-palpable breast carcinomas: correlation of mammographically detected malignant-appearing microcalcifications and molecular prognostic factors. Int J Cancer.

[27] Lee J, Park NJ, Park HY, Kim WW, Kang B, Keum H (2022). Oncologic necessity for the complete removal of residual microcalcifications after neoadjuvant chemotherapy for breast cancer. Sci Rep.

[28] Cheun JH, Lee YJ, Lee JH, Shin Y, Chun JW, Baek SY (2022). Surgical margin status and survival outcomes of breast cancer patients treated with breast-conserving surgery and whole-breast irradiation after neoadjuvant chemotherapy. Breast Cancer Res Treat.

[29] Symmans WF, Peintinger F, Hatzis C, Rajan R, Kuerer H, Valero V (2007). Measurement of residual breast cancer burden to predict survival after neoadjuvant chemotherapy. J Clin Oncol.

[30] Ogston KN, Miller ID, Payne S, Hutcheon AW, Sarkar TK, Smith I (2003). A new histological grading system to assess response of breast cancers to primary chemotherapy: prognostic significance and survival. Breast.

[31] Jeruss JS, Mittendorf EA, Tucker SL, Gonzalez-Angulo AM, Buchholz TA, Sahin AA (2008). Combined use of clinical and pathologic staging variables to define outcomes for breast cancer patients treated with neoadjuvant therapy. J Clin Oncol.

