# Supplementary material for: Impact of residual microcalcifcations on prognosis after neoadjuvant chemotherapy in breast cancer patients
Source: BMC Womens Health. 2024 Mar 20;24:187. doi: 10.1186/s12905-024-02973-9 (PMC10956337; doi:10.1186/s12905-024-02973-9)
Supplement: Supplementary file 1 — Supplementary Material 1. [file 12905_2024_2973_MOESM1_ESM.docx]

| **Supplementary Table 1** Mammographic findings before and after neoadjuvant chemotherapy | | |  |
| --- | --- | --- | --- |
| **Characteristics** | **Before** | **After** | **P value** |
| Morphology of microcalcifications |  |  | < 0.001 |
| Amorphous | 91 (28.1) | 49 (15.3) |  |
| Coarse heterogeneous | 37 (11.3) | 52 (16.1) |  |
| Pleomorphic | 126 (39.3) | 148 (45.7) |  |
| Fine linear/linear branching | 69 (21.3) | 74 (22.9) |  |
| Distribution of microcalcifications |  |  | 0.146 |
| Regional | 70 (21.6) | 56 (17.1) |  |
| Grouped | 104 (32.2) | 116 (36.0) |  |
| Linear | 10 (3.1) | 14 (4.4) |  |
| Segmental | 123 (38.3) | 123 (38.2) |  |
| Diffuse | 16 (4.9) | 14 (4.4) |  |
| Multifocality/multicentricity of microcalcifications |  |  | 0.376 |
| Single | 213 (65.9) | 217 (67.2) |  |
| Multiple | 110 (34.1) | 106 (32.8) |  |
| Extent of microcalcifications, mm | 41.0 (25.0-66.5) | 43.0 (22.0-70.0) | 0.500 |
| Breast density |  |  | 0.163 |
| Almost entirely fatty | 7 (2.2) | 4 (1.2) |  |
| Scattered areas of fibroglandular tissue | 49 (15.2) | 50 (15.5) |  |
| Heterogeneously dense | 157 (48.5) | 181 (56.0) |  |
| Extremely dense | 110 (34.1) | 88 (27.3) |  |
| Change in extent of microcalcifications |  |  |  |
| Decreased |  | 74 (23.0) |  |
| No change |  | 221 (68.4) |  |
| Increased |  | 18 (5.6) |  |
| New |  | 10 (3.0) |  |
| Change in morphology of microcalcifications |  |  |  |
| No change |  | 234 (72.5) |  |
| Change |  | 89 (27.5) |  |
| Data are presented as median (interquartile range) or number (%). | | |  |
