# Supplementary material for: Impact of residual microcalcifcations on prognosis after neoadjuvant chemotherapy in breast cancer patients
Source: BMC Womens Health. 2024 Mar 20;24:187. doi: 10.1186/s12905-024-02973-9 (PMC10956337; doi:10.1186/s12905-024-02973-9)
Supplement: Supplementary file 2 — Supplementary Material 2. [file 12905_2024_2973_MOESM2_ESM.docx]

| **Supplementary Table 2** Changes in mammographic and MRI characteristics after NAC according to breast pathologic response. | | | |
| --- | --- | --- | --- |
| **Characteristics** | **pCR**  **(n=102)** | **Non-pCR**  **(n=221)** | **P value** |
| Change in extent of microcalcifications |  |  | 0.178 |
| Decreased | 22 (21.7) | 48 (21.7) |  |
| No change | 68 (66.7) | 140 (63.4) |  |
| Increased | 1 (0.9) | 16 (7.2) |  |
| New | 3 (2.9) | 6 (2.7) |  |
| Not specified | 8 (7.8) | 11 (5.0) |  |
| Change in morphology of microcalcifications |  |  | 0.819 |
| No change | 46 (45.1) | 104 (47.1) |  |
| Change | 20 (19.6) | 37 (16.7) |  |
| Not specified | 36 (35.3) | 80 (36.2) |  |
| Change in mammographic density |  |  | 0.448 |
| No change | 36 (35.3) | 63 (28.5) |  |
| Change | 29 (28.4) | 73 (33.0) |  |
| Not specified | 37 (36.3) | 85 (38.5) |  |
| Change in size of tumor based on MRI |  |  | <0.001 |
| CR | 50 (49.0) | 24 (10.9) |  |
| PR | 42 (41.2) | 153 (69.2) |  |
| SD | 9 (8.8) | 21 (9.5) |  |
| PD | 0 (0) | 3 (1.4) |  |
| Data are presented as number (%).  Abbreviations: NAC, neoadjuvant chemotherapy; pCR, pathologic complete response; CR, complete response; PR, partial response; SD, stable disease; PD, progressive disease. | | | |
