# Supplementary material for: Impact of residual microcalcifcations on prognosis after neoadjuvant chemotherapy in breast cancer patients
Source: BMC Womens Health. 2024 Mar 20;24:187. doi: 10.1186/s12905-024-02973-9 (PMC10956337; doi:10.1186/s12905-024-02973-9)
Supplement: Supplementary file 3 — Supplementary Material 3. [file 12905_2024_2973_MOESM3_ESM.docx]

| **Supplementary TABLE 3** Univariate and multivariate analyses of breast pathologic complete response (ypT0 or ypTis). | | | | | |
| --- | --- | --- | --- | --- | --- |
| **Characteristics** | **Univariate** | |  | **Multivariate** | |
|  | **Odds ratio (95% CI)** | **P value** |  | **Odds ratio (95% CI)** | **P value** |
| Age at diagnosis, years (ref= <40) |  |  |  |  |  |
| ≥40 | 1.538 (0.868-2.724) | 0.140 |  |  |  |
| HER2^+^(ref= negative) |  |  |  |  |  |
| Positive | 3.046 (1.475-6.290) | 0.003 |  | 2.465 (1.128-5.385) | 0.024 |
| Pre-NAC tumor size on MRI, mm (ref= ≥10) |  |  |  |  |  |
| <10 | 2.158 (0.134-34.855) | 0.588 |  |  |  |
| Post-NAC tumor size on MRI, mm (ref= ≥10) |  |  |  |  |  |
| <10 | 5.091 (2.900-8.939) | <0.001 |  | 2.001 (0.932-4.298) | 0.075 |
| Pre-NAC extent of microcalcifications, mm (ref=<10) |  |  |  |  |  |
| ≥10 | 1.507 (0.412-5.511) | 0.535 |  |  |  |
| Post-NAC extent of microcalcifications, mm (ref= <10) |  |  |  |  |  |
| ≥10 | 2.206 (0.686-7.088) | 0.184 |  |  |  |
| Radiologic response of breast tumor on MRI (ref=PD) |  |  |  |  |  |
| PR | 5.490 (0.716-42.104) | 0.101 |  | 2.467 (0.305-19.932) | 0.397 |
| SD | 8.571 (0.994-73.941) | 0.051 |  | 4.019 (0.441-36.611) | 0.217 |
| CR | 41.667 (5.276-329.047) | <0.001 |  | 7.526 (0.815-69.468) | 0.075 |
| Extent of surgery (ref=total mastectomy) |  |  |  |  |  |
| Partial mastectomy | 2.824 (1.734-4.598) | <0.001 |  | 1.770 (0.996-3.143) | 0.051 |
| Change in extent of microcalcifications (ref= Increased) |  |  |  |  |  |
| No change | 7.771 (1.010-59.824) | 0.059 |  | 6.894 (0.790-60.183) | 0.081 |
| Decreased | 7.333 (0.914-58.838) | 0.061 |  | 7.283 (0.781-67.910) | 0.082 |
| Change in morphology of microcalcifications (ref=no change) |  |  |  |  |  |
| Change | 1.222 (0.641-2.330) | 0.542 |  |  |  |
| Abbreviations: CI, confidence interval; HER2, human epidermal growth factor receptor 2; NAC, neoadjuvant chemotherapy; N/A, not applicable; CR, complete response; PR, partial response; ref, reference; SD, stable disease; PD, progressive disease. | | | | | |
