# Supplementary material for: Impact of residual microcalcifcations on prognosis after neoadjuvant chemotherapy in breast cancer patients
Source: BMC Womens Health. 2024 Mar 20;24:187. doi: 10.1186/s12905-024-02973-9 (PMC10956337; doi:10.1186/s12905-024-02973-9)
Supplement: Supplementary file 4 — Supplementary Material 4. [file 12905_2024_2973_MOESM4_ESM.docx]

**Supplementary Table 4** Incidence of five-year outcomes according to residual microcalcifications and pathologic response

|  | **Non-pCR_w/mic_** | **Non-pCR_w/o mic_** | **pCR_w/ mic_** | **pCR_w/o mic_** |
| --- | --- | --- | --- | --- |
| LFS | 76.9 (60.9- 97.1) | 84.5 (79.4- 89.9) | N/A | 96.4 (92.4-100.0) |
| RFS | 85.7 (71.9- 100.0) | 81.6 (76.2- 87.3) | N/A | 96.5 (92.7- 100.0) |
| DMFS | 73.4 (57.3- 94.2) | 73.7 (67.7- 80.3) | 100.0 (63.3- 100.0) | 96.6 (92.9- 100.0) |
| OS | 86.5 (73.4- 100.0) | 85.7 (80.8- 90.8) | N/A | 96.4 (92.4- 100.0) |

Values are presented as number (95% CI).

Abbreviations: DMFS, distant metastasis-free survival; LFS, local relapse-free survival; N/A, not applicable; OS, overall survival; RFS, regional relapse-free survival.
